# Commensal microbiota modulates larval foraging behaviour, development rate and pupal production in *Bactrocera tryoni*

**DOI:** 10.1186/s12866-019-1648-7

**Published:** 2019-12-24

**Authors:** Juliano Morimoto, Binh Nguyen, Shabnam T. Tabrizi, Ida Lundbäck, Phillip W. Taylor, Fleur Ponton, Toni A. Chapman

**Affiliations:** 1 Department of Biological Sciences, Macquarie University, North Ryde, NSW 2109 Australia; 2 Programa de Pós-Graduação em Ecologia e Conservação, Federal University of Paraná, Curitiba, 19031, CEP: 81531-990 Brazil; 3 The Elizabeth Macarthur Agricultural Institute, New South Wales Department of Primary Industries, Meneagle, NSW 2568 Australia

**Keywords:** Nutrition, Larval behaviour, Development, Microbiota

## Abstract

**Backround:**

Commensal microbes can promote survival and growth of developing insects, and have important fitness implications in adulthood. Insect larvae can acquire commensal microbes through two main routes: by vertical acquisition from maternal deposition of microbes on the eggshells and by horizontal acquisition from the environment where the larvae develop. To date, however, little is known about how microbes acquired through these different routes interact to shape insect development. In the present study, we investigated how vertically and horizontally acquired microbiota influence larval foraging behaviour, development time to pupation and pupal production in the Queensland fruit fly (‘Qfly’), *Bactrocera tryoni*.

**Results:**

Both vertically and horizontally acquired microbiota were required to maximise pupal production in Qfly. Moreover, larvae exposed to both vertically and horizontally acquired microbiota pupated sooner than those exposed to no microbiota, or only to horizontally acquired microbiota. Larval foraging behaviour was also influenced by both vertically and horizontally acquired microbiota. Larvae from treatments exposed to neither vertically nor horizontally acquired microbiota spent more time overall on foraging patches than did larvae of other treatments, and most notably had greater preference for diets with extreme protein or sugar compositions.

**Conclusion:**

The integrity of the microbiota early in life is important for larval foraging behaviour, development time to pupation, and pupal production in Qflies. These findings highlight the complexity of microbial relations in this species, and provide insights to the importance of exposure to microbial communities during laboratory- or mass-rearing of tephritid fruit flies.

## Background

Communities of commensal microorganisms (‘microbiota’) influence a wide variety of behavioural and physiological traits in their animal hosts [1, 2]. The effects of the microbiota on modulation of blood pressure, diabetes and obesity risks have been shown in vertebrates [3, 4], while the microbiota is known to play numerous fitness-associated roles in a vast diversity of invertebrate hosts [5], from changes in developmental rate, nutrition, reproduction, to kin recognition [6–10] and even mate choice, although with conflicting findings [11, 12]. The microbiota can influence host physiology and behaviour at various developmental stages. Host-microbiota interactions are for instance highly influential at the larval stage in insects [13–17]. In the Fritillary butterfly, *Melitaea cinxia*, the gut microbiota is a key determinant of larval growth rate [18]. In mosquitoes, the lack of gut microbiota results in significantly delayed development and reduced likelihood of larvae developing through to adulthood [19, 20].

The microbiota is often composed by a mix of microbes that have co-evolved with the host and therefore are essential to host survival and fitness (primary obligatory symbionts), as well as transient microbes that have not co-evolved with the host but can nonetheless affect hosts’ fitness (secondary facultative symbionts) [21, 22]. Both primary and secondary microbes are mainly acquired through (i) vertical transmission via maternal surface contamination of the egg and (ii) horizontal acquisition from the environment [22–25]. In insects, females can contaminate the eggshells of their progeny with their own microbiota, which is then ingested by hatching larvae [5, 26–31]. Through development, the maintenance of microbiota depends on ingestion of microbes from the environment, most often from dietary sources ([32–40]; see also [22, 24] for reviews). Larvae of some insect species can even develop foraging preferences for certain microbiota strains that support their development [10, 41, 42]. Yet, there has been little investigation of how vertically and horizontally acquired microbiota shape development and larval behaviour.

In tephritid fruit flies, the microbiota is an important determinant of health and performance in both larvae and adults [16, 25, 38, 43–47], and manipulations of microbiota communities have been suggested as a mean of enhancing the performance of insects produced for sterile insect technique (SIT) programs [48–50]. For instance, supplementing Mediterranean fruit fly (*Ceratitis capitata*) larvae with the bacterium *Enterobacter sp*. improves pupal and adult productivity and reduces development time without affecting other fitness-related traits such as mating competitiveness [49]. In the present study, we ascertained the importance of vertically and horizontally acquired microbiota in the tephritid fruit fly *Bactrocera tryoni* Froggatt (Diptera: Tephritidae) (aka ‘Queensland fruit fly’ or ‘Qfly’). Previous studies in Qflies have demonstrated the presence of both vertical [30] and horizontal [51] acquisition of microbiota. We manipulated the microbiota of Qfly eggs and larvae to generate treatments comprised of sterile eggs from which larvae were reared in either sterile or non-sterile diet, as well as the control, conventional, treatment of non-sterile eggs reared in non-sterile diet. Our approach therefore manipulated both permanent and transient members of the microbiota simultaneously. The effects of microbial exposure were measured on larval foraging behaviour, development time to pupation and pupal production. The present study investigates not only the importance of the microbiota for larval behaviour, development rate and pupal production in Qfly, but also highlights that manipulation of the microbiota communities acquired horizontally or vertically may provide a valuable means of enhancing mass-rearing of this species for SIT programs.

## Methods

### Fly stock and egg collection

Eggs were collected from a laboratory-adapted stock of Qfly (> 17 generations-old). The colony has been maintained in non-overlapping generations in a controlled environment room (humidity 65 ± 5%, temperature 25 ± 0.5 °C) with light cycle of 12 h light: 0.5 h dusk:11 h dark: 0.5 h dawn). Adults were maintained with free-choice diets of hydrolysed yeast (MP Biomedicals, Cat. n^o^ 02103304) and commercial cane sugar (CSR® White Sugar), while larvae were maintained using a ‘standard’ gel-based diet that contains Brewer's yeast (Lallemand LBI2250) [52]; Nipagin used in the gel-based diet was obtained from Southern Biological (Cat no. MC11.2). Eggs were collected in a 300 mL semi-transparent white plastic (LDPE) bottle that had perforations of < 1 mm diameter through which females could insert their ovipositor and deposit eggs. The bottle contained 20 mL of water to maintain high humidity. Females were allowed to oviposit for 2 h, after which eggs were transferred to larval diet.

### Experimental procedures

#### Microbiota manipulation of eggs and larvae

An established protocol was used to manipulate microbial exposure of the eggs and larvae [53]. Briefly, eggs were washed twice in 0.5% Chlorite liquid bleach (Peerless JAL®) for 5 min, followed by one wash in 70% ethanol for 2 min, and three washes in Milli-Q water for 2 min each wash. Using a sterilized brush in a sterile environment, the eggs were then transferred onto either non-sterile standard gel-based diets (‘−/+’ treatment) or standard gel-based diets supplemented with 50 μg/mL (final concentration) of streptomycin and tetracycline (stock solution: 10 mg/ml for both) (Cat no. S6501 and T3258 from Sigma Aldrich®, respectively) (‘−/−’ treatment). Finally, eggs with intact microbiota were washed three times in sterile Milli-Q water for 2 min each wash and placed on standard gel-based diets without antibiotics and in non-sterile environment (‘+/+’ treatment). Antibiotics were dissolved in sterile Milli-Q water in sterile 50 mL tubes to create the stock solutions. The stock solution of tetracycline was warmed to 50 °C to increase solubility. Antibiotics were added to the gel-based diet just before the diet set. To quantify and compare the microbial load of larvae in each treatment, we washed groups of three late 2nd instar larvae 3 times in 80% ethanol for 2 min each wash, followed by 3 washes in PBS buffer for 2 min each wash before homogenising the larvae using Sigma Aldrich® autoclavable plastic pestles (Cat no. Z359947). We plated 30 μL of the homogenate (*N* = 5 replicates per treatment) in de *Man-Rogosa-Sharpe* (Oxoid® MRS, Cat no. CM0361) agar, LB agar (Oxoid® Cat no. 22700025), and *Potato-Dextrose Agar *(PDA) (Oxoid® Cat no. CM0139B) plates (*N = 45* plates), and incubated for 48 h at 26 °C, after which we counted the number of colonies (‘CFU’) in the plates. This approach allowed us to quantify culturable bacterial and fungal components of the microbial community. For this study, we consider ‘vertically acquired microbiota’ as the microbiota that is present in the eggs and ‘horizontally acquired microbiota’ as the microbiota potentially present in the diet and in the surrounding environment. The total CFU *per* replicate *per* larvae was estimated as the sum of colonies in all three plates multiplied by the total volume of homogenate. A non-parametric Kruskal-Wallis test was used to test for differences in CFU counts between treatments. As expected, there was a significant effect of treatment on CFU load of the larvae, in which larvae from treatment +/+ had the highest CFU counts, followed by treatment −/+ with intermediate CFU counts, and treatment −/− with no CFU (Additional file 1).

#### Developmental time until pupation and pupal production

For each treatment, ca. 50 eggs (SE: ± 0.274) were placed at the centre of 50 mL Falcon tubes that contained 15 mL of standard gel-based diet (40 replicate tubes per treatment). The egg count was achieved by adding 4 *μ* L of egg-water solution (expected yield of 50 eggs) into the Falcon tubes and then counting the total number of eggs in each Falcon tube under sterile conditions. This approach was needed to avoid contamination of the eggs and diet by airborne microbes (particularly in the −/− treatment); to standardise the methods, we used this protocol for all treatments. When preparing the tubes, diet was poured while warm, and tubes were tilted until diet set in order to generate more surface area of the diet for the larvae. Excess moisture was allowed to evaporate under sterile conditions after which the tubes were sealed. All treatments were maintained in a controlled environment room (humidity 65 ± 5%, temperature 25 ± 0.5 °C) with 12 h light: 0.5 h dusk:11 h dark: 0.5 h dawn cycle.

For collection of pupae, four 50 mL Falcon tubes in which larvae were developing were inserted through 30 mm diameter holes in the lid of a 1.125 L Decor Tellfresh plastic container (12 cm × 9.5 cm × 10.5 cm) so that the top protruded into the plastic container (*N =* 10 replicates per treatment). The plastic containers were sterilized with 70% ethanol, and contained ca. 50 g of autoclaved vermiculite, and laid on their side so that larvae could easily exit from the Falcon tubes to pupate in the vermiculite. No larvae remained in the Falcon tubes at the end of the experiment. This design allowed larvae to pupate in a sterile environment. Pupae were collected by sieving the vermiculite 8, 9 and 10 days after the onset of the experiment, and then holding all collected pupae in 90 mm Petri dishes.

‘Pupal production’ was calculated as the total number of pupae divided by the number of eggs placed on the diet multiplied by 100 (%). ‘Daily pupation percentage’ was measured as the number of pupae collected 8, 9 and 10 days after eggs were placed on the diet divided by the sum of the number of pupae for all days, multiplied by 100 (%). No pupation was observed after 10 days. This allowed us to (1) compare how many pupae were collected each day while standardising for overall pupal production of each treatment group (‘daily pupation percentage’) and (2) identify the day with the highest pupal production (‘peak pupation day’). ANOVA was used to compare treatment groups for pupal production and development time, followed by Student-Newman-Keuls (SNK) posthoc tests. For pupal production, the model contained replicate and treatment as factors in a single model. For developmental time, the model contained replicate, as well as treatment and the linear and quadratic effects of time (and their interactions) as factors in a single model. All statistical analyses were performed using R version 3.4.0 [54]. Figures for developmental time to pupation and pupal production were plotted using the R package ‘ggplot2’ [55].

#### Foraging behaviour

The ratio of yeast-to-sugar (Y:S ratios) from the standard gel-based larval diet [52] was manipulated to create 6 diets (280 mg/mL) with yeast-to-sugar (Y:S) ratios of 1:0, 5:1, 1.5:1, 1:1.6, 1:3.4, and 0:1 (for formulations, see Additional file 2). For the experimental diet mixture, we used hydrolysed yeast obtained from MP Biomedicals (Cat no. 02103304) containing ca. 60% protein according to the product data sheet (Datasheet 02103304). Diets made with hydrolysed yeast are translucent which facilitates the counting of the larvae in the foraging patches during the experiment. Sucrose was obtained from MP Biomedicals (Cat no. 02902978). 20 mL of each diet was poured into 90 mm diameter Petri dishes and allowed to set. In addition to the diets, a 1% agar solution that contained the same components as the diets except for yeast and sugar was prepared; 20 mL of the agar solution was poured to cover a 90 mm diameter Petri dish that was used as the ‘foraging arena’ (*N = 20)*. The pH of all diets, including the agar base of the foraging arena, was adjusted to 3.8–4 using citric acid. After setting and 15 min prior to the onset of the experiment, six equally spaced holes were made around the agar base of the foraging arena by perforating it with a 25 mm diameter plastic tube. The plastic tube and all surfaces were sterilised with Ethanol 80% before use. The same tube was used to cut discs from the experimental diets, which were deposited in the holes in order of increasing Y:S ratio.

Larvae were reared in 50 mL Falcon tubes as described previously (i.e., treatments −/−, −/+. +/+). At 4–5 days after egg collection, 25 late 2nd instar larvae from each treatment were collected with a soft brush and placed at the centre of foraging arenas (7 replicates per treatment), which were then covered to minimize loss of moisture and placed in a dark room to minimise visual stimuli. The number of larvae on each of the discs of diet and on the agar base between discs was assessed 1 h, 2 h, 4 h, 6 h, and 24 h after larvae were placed in the arena. To analyse larval foraging preference, a multinomial logistic regression model was fitted using the ‘multinom’ function of the ‘nnet’ package in R [56] with time, treatment, and their interactions as factors. A multinomial logistic regression measures the relative log-odds of a choice between a reference level (agar base) and a comparative level (each diet).

If relative log-odds > 0, the foraging preference for the diet is higher than to the agar base. If relative log-odds < 0, the foraging preference is higher for the agar base than to the diet. Note that the reference and comparative levels are taken within treatments, that is, the foraging preference for each diet is compared with agar base *within* the treatment. The interaction term measures the statistical significance between two *within* treatment differences in foraging preference for agar base vs. diet. For example, the interaction term measures the difference in relative log-odds of agar base vs. diet 1 within treatment A, and agar base vs. diet 1 within treatment B. The same comparison is applied to all diets. This approach was necessary to account for the non-independence of the data points within each foraging arena over time, and the multiple simultaneous choices of diets presented to the larvae. Statistical inferences of the relative log-odds were made based on the *t-*distribution (α = 0.05). Relative log-odds were plotted in Excel version 14.7.3.

## Results

### The microbiota affects development time and pupal production

**Fig. 1:**
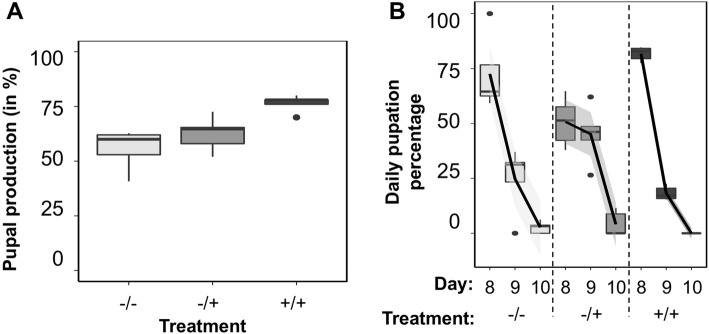
The effects of the microbiota on pupation in Qfly larvae. **a** Pupal production (in %, from 50 ± 0.274 eggs per treatment). **b** Daily pupation percentage from days 8–10 after the onset of the experiment

Manipulation of microbiota significantly affected pupal production (*Treatment:* F_2,11_ = 11.710, *p* = 0.002, Additional file 2: Table S2), whereby more pupae were produced from treatment +/+ than from treatments −/− and −/+ (Fig. 1 a, Additional file 2: Table S2). There was no significant difference between treatments −/− and −/+ on pupal production (Additional file 2: Table S2). There were also significant interactions between the linear and quadratic effects of time (days after egg collection) and treatment on daily pupation percentage (Day * Treatment: F_2,35_ = 8.315, *p* = 0.001, *Day*^*2*^ * Treatment: F_2,35_ = 15.446, *p* < 0.001, Additional file 2: Table S3), whereby treatments −/− and +/+ had a peak in daily pupation percentage on day 8, after which daily pupation percentage declined in day 9 and 10, whereas treatment −/+ had similar daily pupation percentage on days 8 and 9 before declining sharply on day 10 (Fig. 1 b, Additional file 2: Table S3).

### The microbiota affects larval foraging behaviour

**Fig. 2:**
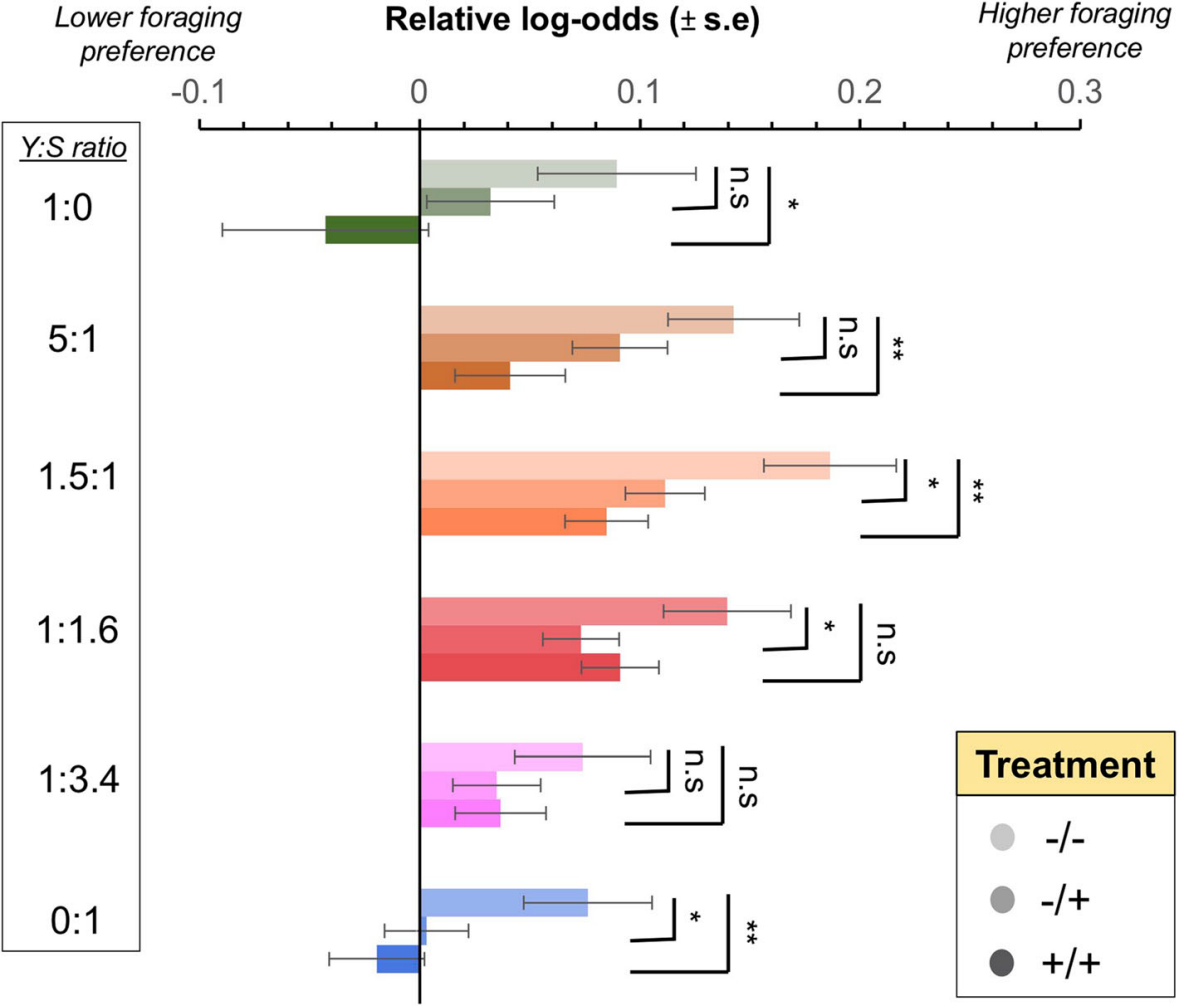
The microbiota modulates larval foraging preference. Relative Log-odds (± standard error) showing larval foraging preference against diets with varying Y:S ratio (25 larvae per replicate, 7 replicates per treatment). Comparisons between treatments were made with −/− treatment as the reference level. * *p* < 0.05; ** 0.001 < *p* < 0.01; n.s. = non-significant. Light palette: treatment −/−; Intermediate palette: treatment −/+; Dark palette: treatment +/+

Larval foraging preference was assessed by offering larvae a choice amongst 6 diets that varied in yeast-to-sugar ratios (Y:S ratios), including diets that were yeast (protein) biased, balanced, or sugar biased. Larvae of treatment −/− had greater preference to forage in extreme Y:S ratios relative to the agar base than did larvae of other treatment groups (see Additional file 2: Table S4). In particular, larvae from treatment −/− had higher foraging preference for diets of Y:S ratio 1:0 (protein biased) and Y:S ratio 0:1 (no protein) (Fig. 2) than did larvae from treatment +/+. Larvae from treatment −/− also displayed significantly higher foraging preference for balanced diets (i.e., Y:S 5:1 and 1.5:1) in comparison to larvae from treatment +/+ (Fig. 2). On the other hand, absence of vertically acquired microbes for larvae on non-sterile diet (i.e., treatment −/+) influenced preference for foraging on balanced and sugar biased diets (Fig. 2 and Additional file 2: Table S4). For instance, treatment −/+ larvae and treatment −/− larvae were significantly different in foraging preference for diets of Y:S 1.5:1, 1:1.6 and 0:1 (Fig. 2, Additional file 2: Table S4). Overall, the foraging preference patterns of larvae from treatments −/+ and +/+ were more similar than to that of larvae from the treatment −/− (Fig. 2).

## Discussion

Host-microbiota interactions are highly influential in larval development and adult fitness of many insect species [13–16, 25, 57, 58]. Here, we showed that in Qfly the microbiota affects developmental time to pupation and pupal production, as well as larval foraging behaviour, particularly preference for foraging on diets with extreme nutrient composition. In tephritids, the microbiota modifies the nutritional environment of the larvae [58] and serves as food for adults [25]. Moreover, manipulations of the gut microbiota have been proposed as means to enhance the performance of sterile adult flies released in SIT programs [48–50] since larval nutrition and health is an important determinant of the yield and quality of mass-reared adults. The present study provides insights to the role of vertically and horizontally acquired bacteria in development and pre-pupal survivorship of Qfly but also provides a starting point for future work aiming at enhancing the quantity and quality of mass-reared Qfly for SIT.

### Effects of the microbiota on pupal production

Our data showed that vertically and horizontally acquired microbiota communities were important for development time and pupal production in Qflies*.* For instance, daily pupation percentage showed a similar pattern of linear decrease over time in treatments −/− and +/+, which was not observed for treatment −/+, suggesting that horizontally acquired microbiota could potentially influence the time until pupation independently of vertically acquired microbiota. It is unclear why larvae from axenic (germ-free) eggs that were exposed to horizontally acquired microbiota (i.e., treatment −/+) showed a delay in pupal production. It is possible that axenic larvae are more susceptible to infection by pathogenic microbes from the environment (see for instance [59–61]) that could have a negative effect on larval development. Despite this, pupal production was significantly lower in treatments −/− and −/+ compared with treatment +/+, revealing that horizontally acquired microbes are insufficient to fully rescue pupal production and highlighting the importance of vertically transmitted microbiota for development. The mechanisms through which the microbiota affect pupal production in Qfly is unknown. It is possible that specific strains of the microbiota regulate factors underpinning life-stage transitions of flies. For example, in *Bactrocera dorsalis* gut bacteria of the genus *Enterococcus* have been found to have positive effects, and *Lactobacillus* to have negative effects, on larval development and pupation [62], but the molecular mechanisms of these effects are not known. In Qflies, two yeast strains, *Pichia kluyveri* and *Hanseniaspora uvarum*, have been recently proved to play an important role in pupal production [43], although it is not certain when and from where these fungi are acquired by larvae. These previous findings suggest a complex interplay between the fungal and bacterial components of the microbiota on development [41–43], and open an important avenue for developing approaches that exploit fungi or bacteria, or both, to enhance development in mass-rearing programs. Our results are in agreement with previous literature showing that the microbiota can promote development to pupation in Qflies [43]*.* It is unlikely that our results were influenced by the sterilization treatment used to remove the microbiota from the eggshells since our findings are broadly consistent with previous literature using axenic (germ-free) models in *Drosophila*, whereby the gut microbiota at early stages of development affects larval development and behaviour, as well as pupal production and adult traits (e.g. [10, 35, 41, 42]), although recently some experimental procedures have been questioned (e.g., [63]).

### Effects of the microbiota on larval foraging behaviour

Bacteria that were vertically and/or horizontally acquired affected Qfly larval foraging behaviour. For instance, the number of larvae on foraging patches, rather than the agar base, was relatively high for treatment −/−, intermediate for treatment −/+, and relatively low for treatment +/+ in comparison with other treatments. These patterns were particularly evident for extreme protein- and sugar-biased diets for which the larvae from treatment −/− exhibited much higher preference than did larvae from treatments −/+ and +/+ (Fig. 2). Together, these findings show that vertically and horizontally acquired microbiota can act in combination to regulate larval foraging behaviour patterns. The exact mechanism through which the microbiota modulates Qfly larval foraging behaviour is unknown, although it is possible that microbes modulate nutrient-specific larval foraging behaviour due to their differential carbohydrate and protein metabolism. For instance, a recent study has shown that the gut microbiota can modulate appetite for amino acids in *D. melanogaster* adults [9], although whether the gut microbiota also modulates amino acid appetite in larvae remains unknown. It is also possible that the absence of microbiota may affect metabolic processes and nutrient assimilation in Qfly larvae, as has been found previously in *D. melanogaster* [41, 42]. The total absence of microbiota (−/− treatment) resulted in Qfly larvae with greater tendency to forage in all diets, including those with extreme nutritional values (e.g., Y:S 0:1). This result might indicate a reduced ability of larvae to discriminate or to balance nutrient intake, and might also suggest a broader nutritional requirement of these larvae compared with larvae that are exposed to vertically and horizontally acquired microbial communities. In addition to influencing larval foraging behaviour, microbiota in the larval diet is also known to alter the diet’s nutritional composition. For instance, the microbiota in the diet increases the amino acid content of the substrate where larvae develop, which in turn may affect how larvae balance their dietary preferences [58]. It remains unknown whether these potential effects of the microbiota on larval foraging preferences are carried through to adulthood. Previous studies have shown that laboratory-adapted adult female Qflies are equally attracted to diets with and without microbiota supplementation, suggesting that the modulation of adult dietary preferences could be independent of the microbiota colonising the diet in adult Qflies [64]. However, to our knowledge, there have been no studies that manipulate the microbiota of adult Qflies (instead of the microbiota of the diet) to investigate changes in adult foraging preferences. Thus, future studies using approaches similar to those of the present study but applied to adults are needed in order to shed light into whether the microbiota-associated changes in foraging preferences at the larval stage are also observed in adults.

## Conclusion

The present study reveals combined effects of vertically and horizontally acquired microbes on development time, pupal production and larval foraging behaviour in Qflies. These findings contribute to the understanding of fitness-related effects of host-microbial interactions, and provide a starting point for future investigations of how microbiota affects early life stages of this species, as well as guiding development of protocols for enhanced large scale rearing for Qfly SIT programs.

## Supplementary information

**Additional file 1.** Manipulation of the microbiota in Qfly larvae. Total CFU counts of Qfly larvae. Kruskal-Wallis *χ* ^2^ = 13.011, df = 2, *p* = 0.0015 (see Main Text). Light grey: −/− treatment; Intermediate grey: −/+ treatment; Dark grey: +/+ treatment. Letters indicate statistically significant differences in pairwise Kruskal-Wallis comparisons.

**Additional file 2: Table S1** Diet information. The recipes for the diets used in this study. **Table S2** Output of the model investigating the effects of the microbiota on pupal production. Bold – *p* < 0.05. **Table S3** Output of the model investigating the effects of the microbiota on developmental time to pupation. Bold – *p* < 0.05. **Table S4** Complete analysis of the multinomial logistic regression investigating the role of microbiota on larvae foraging preference.

## Data Availability

The raw data used in this study are available in the figures and tables and upon direct request to the lead author.

## Abbreviations

CFU: Colony-forming units
Qfly: Queensland fruit fly
SIT: Sterile Insect Technique
Y:S: Yeast:sugar ratio
