# Supplementary figures and images for: Commensal microbiota modulates larval foraging behaviour, development rate and pupal production in Bactrocera tryoni
Source: BMC Microbiol. 2019 Dec 24;19(Suppl 1):286. doi: 10.1186/s12866-019-1648-7 (PMC6929265; doi:10.1186/s12866-019-1648-7)

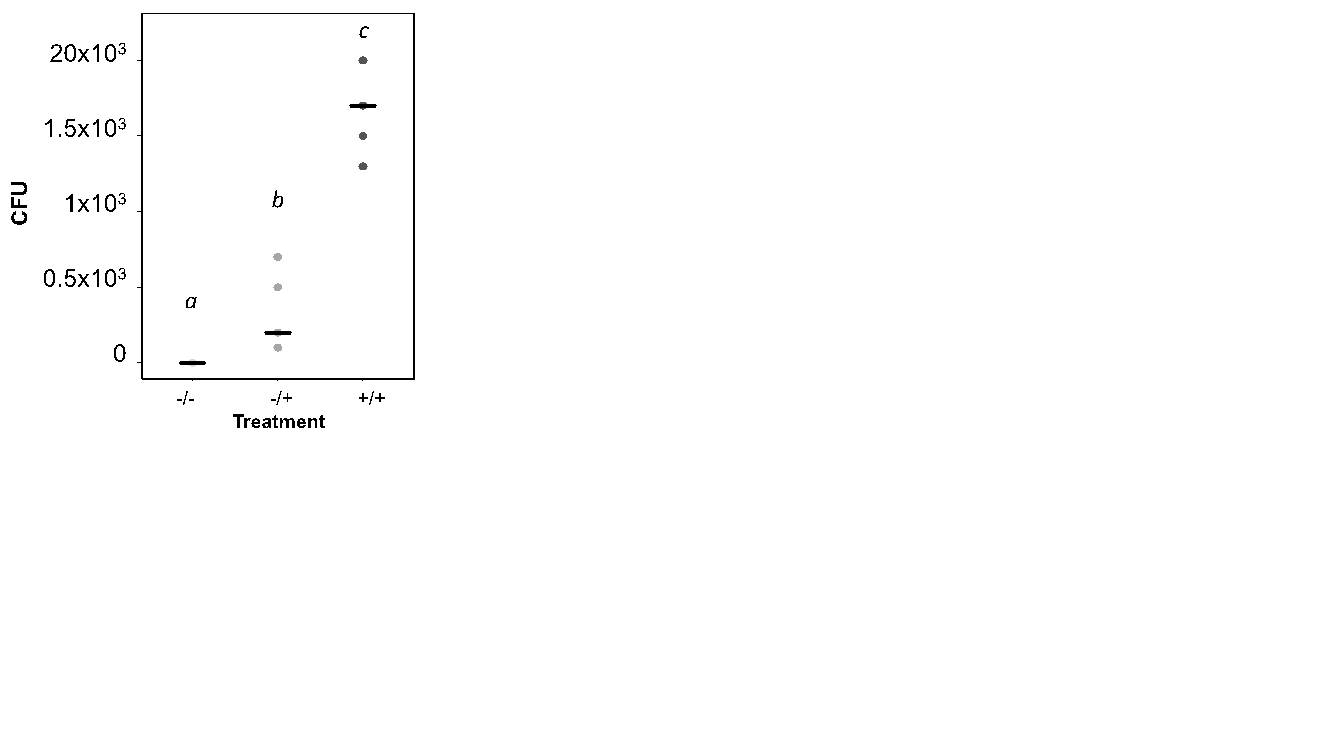

Supplement: Supplementary file 1 — Additional file 1. Manipulation of the microbiota in Qfly larvae. Total CFU counts of Qfly larvae. Kruskal-Wallis χ 2 = 13.011, df = 2, p = 0.0015 (see Main Text). Light grey: −/− treatment; Intermediate grey: −/+ treatment; Dark grey: +/+ treatment. Letters indicate statistically significant differences in pairwise Kruskal-Wallis comparisons. [file 12866_2019_1648_MOESM1_ESM.png]
